# [(4*S*,5*S*)-2,2-Dimethyl-1,3-dioxolane-4,5-di­yl]bis­[*N*-(thio­phen-2-yl­methyl­idene)methanamine]

**DOI:** 10.1107/S1600536812001298

**Published:** 2012-01-18

**Authors:** Yan Jiang, Jing Bian, Xiaoqiang Sun

**Affiliations:** a Key Laboratory of Fine Chemical Engineering, Changzhou University, Changzhou 213164, Jiangsu, People’s Republic of China

## Abstract

In the title compound, C_17_H_20_N_2_O_2_S_2_, the five-membered heterocycle exhibits an envelope conformation and the mol­ecular chirality and configuration are well preserved from l-tartaric acid. The dihedral angle between the two thio­phene rings is 17.0 (2)°. In the crystal, mol­ecules are linked by C—H⋯O and C—H⋯S hydrogen inter­actions, which are effective in the stabilization of the crystal structure.

## Related literature

For general background to spiranes, see: Takashi *et al.* (2011 ▶); Yong (2001 ▶).
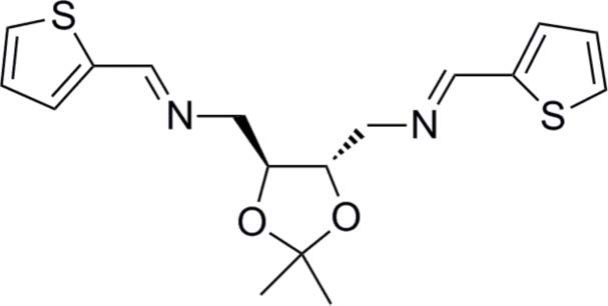


## Experimental

### 

#### Crystal data

C_17_H_20_N_2_O_2_S_2_
*M* *_r_* = 348.47
Monoclinic, 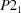

*a* = 10.475 (2) Å
*b* = 7.4792 (15) Å
*c* = 11.533 (2) Å
β = 92.339 (4)°
*V* = 902.8 (3) Å^3^
*Z* = 2
Mo *K*α radiation
μ = 0.31 mm^−1^
*T* = 296 K
0.20 × 0.18 × 0.15 mm

#### Data collection

Bruker SMART CCD area-detector diffractometer
Absorption correction: multi-scan (*SADABS*; Sheldrick, 2004 ▶) *T*_min_ = 0.942, *T*_max_ = 0.956
5249 measured reflections
3140 independent reflections
2575 reflections with *I* > 2σ(*I*)
*R*_int_ = 0.043

#### Refinement

*R*[*F* ^2^ > 2σ(*F* ^2^)] = 0.038
*wR*(*F* ^2^) = 0.092
*S* = 1.02
3140 reflections
211 parameters
19 restraints
H-atom parameters constrained
Δρ_max_ = 0.14 e Å^−3^
Δρ_min_ = −0.18 e Å^−3^
Absolute structure: Flack (1983 ▶), 1322 Friedel pairs
Flack parameter: 0.00 (8)

### 

Data collection: *SMART* (Bruker, 2000 ▶); cell refinement: *SAINT* (Bruker, 2000 ▶); data reduction: *SAINT*; program(s) used to solve structure: *SHELXTL* (Sheldrick, 2008 ▶); program(s) used to refine structure: *SHELXTL*; molecular graphics: *SHELXTL*; software used to prepare material for publication: *SHELXTL*.

## Supplementary Material

Crystal structure: contains datablock(s) I, global. DOI: 10.1107/S1600536812001298/fk2048sup1.cif

Structure factors: contains datablock(s) I. DOI: 10.1107/S1600536812001298/fk2048Isup2.hkl

Supplementary material file. DOI: 10.1107/S1600536812001298/fk2048Isup3.cml

Additional supplementary materials:  crystallographic information; 3D view; checkCIF report

## Figures and Tables

**Table 1:** Hydrogen-bond geometry (Å, °)

*D*—H⋯*A*	*D*—H	H⋯*A*	*D*⋯*A*	*D*—H⋯*A*
C9—H9⋯O1^i^	0.93	2.56	3.431 (4)	155
C8—H8⋯S2^i^	0.93	2.94	3.793 (3)	153
C12—H12⋯O1^ii^	0.93	2.68	3.466 (4)	143

Symmetry codes: (i) 
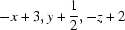
; (ii) 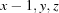.

**Fig. 1.**
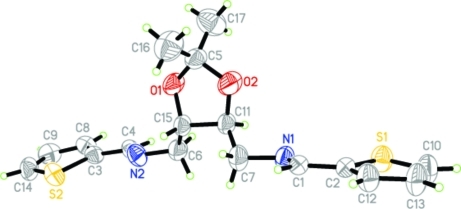
The molecular structure of the title compound. Displacement ellipsoids are drawn at the 50% probability level.

**Fig. 2.**
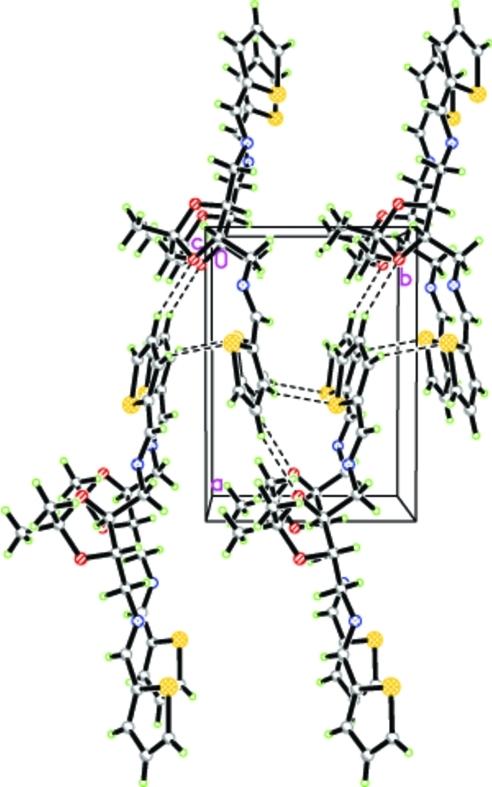
Crystal packing viewed along the c axis. Dashed lines indicate C—H···O and C—H···S interactions.

## supplementary crystallographic information

### Comment

Multidentate and chiral C*_2_*-symmetric ligands have attracted considerable interest, however, the number of chiral precursors available from nature is seriously limited (Yong, 2001). The *L*-(+)-tartaric acid is a well known chiral pool possessing two useful chiral centers which is an important chiral material in synthesis (Takashi *et al.*, 2011). Herein, we synthesized ((4*S*,5*S*)-2,2-dimethyl-1,3- dioxolane-4,5-diyl)bis(*N*-(thiophen-2-ylmethylene)methanamine) based on *L*-tartaric acid and present the structure of it. The five-membered heterocycle (Fig. 1) adopts envelope conformation, the molecular chirality and configuration are well preserved from *L*-tartaric acid. The dihedral angle between the two thiofuran rings is 17.0 (2)°. Molecules are linked by intermolecular weak hydrogen interactions (C—H···O and C—H···S) and probabely C—H···π interactions which are effective in the stabilization of the crystal structure. Fig. 2 shows the crystal packing of the title compound along the *c* axis.

### Experimental

To a solution of 2-thiophenealdehyde (0.9 g, 8.04 mmol) in ethanol (10 ml), ((4*S*,5*S*)-2,2-dimethyl-1,3-dioxolane-4,5-diyl)dimethanamine (0.6 g, 3.75 mmol) dissolved in ethanol (10 ml) was added. The mixture was refluxed for 2 h to complete the reaction and then cooled to room temperature. The compound was recrystallized from ethanol to afford a yellow solid (1 g, 76% yield, m.p. 361.5–363.4 K). Single crystals suitable for X-ray diffraction were also obtained by evaporation of an ethanol solution.

### Refinement

All H atoms were placed in geometrically idealized positions and constrained to ride on their parent atoms, with C—H distances of 0.93–0.98 Å, and with *U*_iso_(H) = 1.2(1.5 for methyl)*U*_eq_(C). As the Flack parameter was not unambiguous the data were refined using TWIN and BASF.

### Crystal data

**Table:**

C_17_H_20_N_2_O_2_S_2_	*F*(000) = 368
*M**_r_* = 348.47	*D*_x_ = 1.282 Mg m^−^^3^
Monoclinic, *P*2_1_	Mo *K*α radiation, λ = 0.71073 Å
Hall symbol: P 2yb	Cell parameters from 1762 reflections
*a* = 10.475 (2) Å	θ = 2.6–24.3°
*b* = 7.4792 (15) Å	µ = 0.31 mm^−^^1^
*c* = 11.533 (2) Å	*T* = 296 K
β = 92.339 (4)°	Block, colourless
*V* = 902.8 (3) Å^3^	0.20 × 0.18 × 0.15 mm
*Z* = 2	

### Data collection

**Table:**

Bruker SMART CCD area-detector diffractometer	3140 independent reflections
Radiation source: fine-focus sealed tube	2575 reflections with *I* > 2σ(*I*)
graphite	*R*_int_ = 0.043
phi and ω scans	θ_max_ = 25.5°, θ_min_ = 1.8°
Absorption correction: multi-scan (*SADABS*; Sheldrick, 2004)	*h* = −8→12
*T*_min_ = 0.942, *T*_max_ = 0.956	*k* = −9→8
5249 measured reflections	*l* = −13→13

### Refinement

**Table:**

Refinement on *F*^2^	Secondary atom site location: difference Fourier map
Least-squares matrix: full	Hydrogen site location: inferred from neighbouring sites
*R*[*F*^2^ > 2σ(*F*^2^)] = 0.038	H-atom parameters constrained
*wR*(*F*^2^) = 0.092	*w* = 1/[σ^2^(*F*_o_^2^) + (0.0442*P*)^2^] where *P* = (*F*_o_^2^ + 2*F*_c_^2^)/3
*S* = 1.02	(Δ/σ)_max_ < 0.001
3140 reflections	Δρ_max_ = 0.14 e Å^−^^3^
211 parameters	Δρ_min_ = −0.18 e Å^−^^3^
19 restraints	Absolute structure: Flack (1983), 1322 Friedel pairs
Primary atom site location: structure-invariant direct methods	Flack parameter: 0.00 (8)

### Special details

**Table:**

Geometry. All e.s.d.'s (except the e.s.d. in the dihedral angle between two l.s. planes) are estimated using the full covariance matrix. The cell e.s.d.'s are taken into account individually in the estimation of e.s.d.'s in distances, angles and torsion angles; correlations between e.s.d.'s in cell parameters are only used when they are defined by crystal symmetry. An approximate (isotropic) treatment of cell e.s.d.'s is used for estimating e.s.d.'s involving l.s. planes.
Refinement. Refinement of *F*^2^ against ALL reflections. The weighted *R*-factor *wR* and goodness of fit *S* are based on *F*^2^, conventional *R*-factors *R* are based on *F*, with *F* set to zero for negative *F*^2^. The threshold expression of *F*^2^ > σ(*F*^2^) is used only for calculating *R*-factors(gt) *etc*. and is not relevant to the choice of reflections for refinement. *R*-factors based on *F*^2^ are statistically about twice as large as those based on *F*, and *R*- factors based on ALL data will be even larger.

### Fractional atomic coordinates and isotropic or equivalent isotropic displacement parameters (Å^2^)

**Table:**

	*x*	*y*	*z*	*U*_iso_*/*U*_eq_	
S1	0.51034 (7)	0.34500 (12)	0.54421 (6)	0.0557 (2)	
S2	1.38990 (7)	0.13103 (10)	1.18352 (6)	0.0563 (2)	
C1	0.5853 (2)	0.1387 (4)	0.7331 (2)	0.0410 (6)	
H1	0.5679	0.0569	0.7911	0.049*	
C2	0.4830 (2)	0.1972 (4)	0.6549 (2)	0.0401 (6)	
N1	0.6976 (2)	0.1954 (3)	0.72485 (19)	0.0479 (6)	
C3	1.4097 (2)	0.2230 (4)	1.0493 (2)	0.0437 (6)	
O1	1.10405 (16)	−0.0651 (3)	0.77918 (17)	0.0565 (5)	
C4	1.3054 (3)	0.2405 (4)	0.9630 (2)	0.0471 (7)	
H4	1.3210	0.3027	0.8953	0.056*	
N2	1.1964 (2)	0.1774 (4)	0.97401 (19)	0.0530 (6)	
C5	1.0211 (3)	−0.1649 (4)	0.7025 (2)	0.0537 (7)	
C6	1.1023 (3)	0.2134 (4)	0.8812 (3)	0.0531 (7)	
H6A	1.1456	0.2609	0.8151	0.064*	
H6B	1.0440	0.3046	0.9069	0.064*	
C7	0.7933 (3)	0.1333 (5)	0.8120 (2)	0.0567 (8)	
H7A	0.7628	0.0256	0.8487	0.068*	
H7B	0.8061	0.2240	0.8715	0.068*	
C8	1.5345 (3)	0.2735 (4)	1.0366 (2)	0.0495 (7)	
H8	1.5644	0.3257	0.9698	0.059*	
C9	1.6117 (3)	0.2371 (5)	1.1363 (3)	0.0608 (8)	
H9	1.6984	0.2638	1.1430	0.073*	
C10	0.3523 (3)	0.3486 (6)	0.5036 (3)	0.0653 (8)	
H10	0.3180	0.4158	0.4419	0.078*	
C11	0.9188 (2)	0.0949 (4)	0.7565 (2)	0.0457 (7)	
H11	0.9426	0.1971	0.7088	0.055*	
O2	0.90851 (19)	−0.0604 (3)	0.6865 (2)	0.0779 (7)	
C12	0.3575 (3)	0.1538 (5)	0.6571 (2)	0.0538 (7)	
H12	0.3242	0.0739	0.7097	0.065*	
C13	0.2831 (3)	0.2434 (5)	0.5706 (3)	0.0670 (9)	
H13	0.1949	0.2306	0.5612	0.080*	
C14	1.5472 (3)	0.1603 (5)	1.2200 (3)	0.0615 (9)	
H14	1.5845	0.1262	1.2912	0.074*	
C15	1.0267 (2)	0.0527 (4)	0.8430 (2)	0.0453 (7)	
H15	0.9937	−0.0092	0.9105	0.054*	
C16	0.9858 (4)	−0.3401 (5)	0.7552 (4)	0.0851 (11)	
H16A	0.9536	−0.3199	0.8308	0.128*	
H16B	1.0599	−0.4155	0.7617	0.128*	
H16C	0.9212	−0.3972	0.7067	0.128*	
C17	1.0853 (3)	−0.1869 (6)	0.5889 (3)	0.0855 (12)	
H17A	1.0303	−0.2529	0.5358	0.128*	
H17B	1.1642	−0.2504	0.6016	0.128*	
H17C	1.1022	−0.0712	0.5568	0.128*	

### Atomic displacement parameters (Å^2^)

**Table:**

	*U*^11^	*U*^22^	*U*^33^	*U*^12^	*U*^13^	*U*^23^
S1	0.0541 (4)	0.0605 (5)	0.0525 (4)	0.0002 (4)	0.0023 (3)	0.0154 (4)
S2	0.0611 (5)	0.0610 (5)	0.0466 (4)	−0.0102 (4)	−0.0018 (3)	0.0023 (4)
C1	0.0447 (15)	0.0404 (15)	0.0378 (13)	0.0071 (13)	0.0013 (11)	−0.0003 (13)
C2	0.0392 (14)	0.0417 (15)	0.0394 (13)	0.0054 (12)	0.0011 (11)	−0.0008 (12)
N1	0.0394 (13)	0.0531 (16)	0.0507 (13)	0.0081 (11)	−0.0034 (10)	0.0050 (11)
C3	0.0493 (16)	0.0386 (15)	0.0428 (14)	−0.0064 (13)	−0.0028 (12)	−0.0072 (12)
O1	0.0404 (10)	0.0560 (13)	0.0717 (12)	0.0093 (9)	−0.0144 (9)	−0.0117 (11)
C4	0.0574 (18)	0.0430 (16)	0.0401 (14)	−0.0056 (14)	−0.0061 (13)	−0.0031 (13)
N2	0.0486 (13)	0.0649 (18)	0.0445 (12)	−0.0056 (12)	−0.0102 (10)	−0.0034 (12)
C5	0.0486 (15)	0.0469 (17)	0.0646 (17)	0.0053 (15)	−0.0102 (13)	−0.0117 (16)
C6	0.0515 (16)	0.0516 (19)	0.0553 (17)	0.0053 (14)	−0.0082 (14)	−0.0019 (15)
C7	0.0461 (16)	0.072 (2)	0.0519 (16)	0.0054 (16)	−0.0050 (13)	0.0108 (17)
C8	0.0515 (18)	0.0472 (17)	0.0497 (16)	−0.0113 (13)	0.0018 (14)	−0.0049 (14)
C9	0.0450 (16)	0.062 (2)	0.075 (2)	−0.0085 (15)	−0.0073 (16)	−0.0150 (18)
C10	0.0618 (19)	0.077 (2)	0.0566 (17)	0.008 (2)	−0.0081 (15)	0.020 (2)
C11	0.0415 (14)	0.0458 (17)	0.0489 (14)	0.0037 (12)	−0.0092 (12)	−0.0010 (12)
O2	0.0607 (13)	0.0720 (16)	0.0975 (16)	0.0196 (12)	−0.0402 (12)	−0.0357 (14)
C12	0.0469 (15)	0.063 (2)	0.0516 (16)	−0.0044 (15)	0.0005 (13)	0.0115 (16)
C13	0.0433 (17)	0.084 (3)	0.072 (2)	0.0022 (17)	−0.0138 (16)	0.015 (2)
C14	0.0631 (19)	0.061 (2)	0.0581 (18)	0.0014 (17)	−0.0218 (15)	0.0003 (17)
C15	0.0390 (14)	0.0486 (16)	0.0476 (15)	0.0041 (13)	−0.0089 (12)	0.0022 (14)
C16	0.086 (2)	0.061 (2)	0.108 (3)	−0.010 (2)	−0.003 (2)	0.004 (2)
C17	0.073 (2)	0.106 (4)	0.077 (2)	−0.012 (2)	0.0019 (19)	−0.017 (2)

### Geometric parameters (Å, °)

**Table:**

S1—C10	1.703 (3)	C7—H7A	0.9700
S1—C2	1.721 (3)	C7—H7B	0.9700
S2—C14	1.699 (3)	C8—C9	1.404 (4)
S2—C3	1.714 (3)	C8—H8	0.9300
C1—N1	1.258 (3)	C9—C14	1.331 (5)
C1—C2	1.440 (3)	C9—H9	0.9300
C1—H1	0.9300	C10—C13	1.337 (5)
C2—C12	1.355 (4)	C10—H10	0.9300
N1—C7	1.466 (3)	C11—O2	1.417 (3)
C3—C8	1.375 (4)	C11—C15	1.510 (3)
C3—C4	1.453 (3)	C11—H11	0.9800
O1—C15	1.422 (3)	C12—C13	1.410 (4)
O1—C5	1.425 (3)	C12—H12	0.9300
C4—N2	1.247 (4)	C13—H13	0.9300
C4—H4	0.9300	C14—H14	0.9300
N2—C6	1.450 (3)	C15—H15	0.9800
C5—O2	1.421 (3)	C16—H16A	0.9600
C5—C16	1.497 (5)	C16—H16B	0.9600
C5—C17	1.505 (4)	C16—H16C	0.9600
C6—C15	1.496 (4)	C17—H17A	0.9600
C6—H6A	0.9700	C17—H17B	0.9600
C6—H6B	0.9700	C17—H17C	0.9600
C7—C11	1.513 (4)		
			
C10—S1—C2	91.42 (15)	C14—C9—H9	123.7
C14—S2—C3	91.17 (15)	C8—C9—H9	123.7
N1—C1—C2	121.6 (3)	C13—C10—S1	112.0 (2)
N1—C1—H1	119.2	C13—C10—H10	124.0
C2—C1—H1	119.2	S1—C10—H10	124.0
C12—C2—C1	127.8 (3)	O2—C11—C15	104.0 (2)
C12—C2—S1	111.0 (2)	O2—C11—C7	110.4 (2)
C1—C2—S1	121.2 (2)	C15—C11—C7	113.6 (2)
C1—N1—C7	117.2 (2)	O2—C11—H11	109.5
C8—C3—C4	126.4 (3)	C15—C11—H11	109.5
C8—C3—S2	110.9 (2)	C7—C11—H11	109.5
C4—C3—S2	122.6 (2)	C11—O2—C5	109.5 (2)
C15—O1—C5	107.45 (19)	C2—C12—C13	112.4 (3)
N2—C4—C3	124.2 (3)	C2—C12—H12	123.8
N2—C4—H4	117.9	C13—C12—H12	123.8
C3—C4—H4	117.9	C10—C13—C12	113.1 (3)
C4—N2—C6	116.9 (2)	C10—C13—H13	123.5
O2—C5—O1	105.9 (2)	C12—C13—H13	123.5
O2—C5—C16	108.4 (3)	C9—C14—S2	113.0 (2)
O1—C5—C16	111.2 (3)	C9—C14—H14	123.5
O2—C5—C17	110.2 (3)	S2—C14—H14	123.5
O1—C5—C17	108.3 (3)	O1—C15—C6	110.1 (2)
C16—C5—C17	112.6 (3)	O1—C15—C11	102.4 (2)
N2—C6—C15	113.8 (2)	C6—C15—C11	113.5 (2)
N2—C6—H6A	108.8	O1—C15—H15	110.2
C15—C6—H6A	108.8	C6—C15—H15	110.2
N2—C6—H6B	108.8	C11—C15—H15	110.2
C15—C6—H6B	108.8	C5—C16—H16A	109.5
H6A—C6—H6B	107.7	C5—C16—H16B	109.5
N1—C7—C11	110.6 (2)	H16A—C16—H16B	109.5
N1—C7—H7A	109.5	C5—C16—H16C	109.5
C11—C7—H7A	109.5	H16A—C16—H16C	109.5
N1—C7—H7B	109.5	H16B—C16—H16C	109.5
C11—C7—H7B	109.5	C5—C17—H17A	109.5
H7A—C7—H7B	108.1	C5—C17—H17B	109.5
C3—C8—C9	112.2 (3)	H17A—C17—H17B	109.5
C3—C8—H8	123.9	C5—C17—H17C	109.5
C9—C8—H8	123.9	H17A—C17—H17C	109.5
C14—C9—C8	112.7 (3)	H17B—C17—H17C	109.5
			
N1—C1—C2—C12	−176.7 (3)	N1—C7—C11—C15	172.7 (3)
N1—C1—C2—S1	1.3 (4)	C15—C11—O2—C5	−16.5 (3)
C10—S1—C2—C12	0.7 (3)	C7—C11—O2—C5	−138.8 (3)
C10—S1—C2—C1	−177.6 (2)	O1—C5—O2—C11	−3.2 (3)
C2—C1—N1—C7	177.3 (2)	C16—C5—O2—C11	116.2 (3)
C14—S2—C3—C8	−0.2 (2)	C17—C5—O2—C11	−120.1 (3)
C14—S2—C3—C4	179.2 (3)	C1—C2—C12—C13	176.8 (3)
C8—C3—C4—N2	172.8 (3)	S1—C2—C12—C13	−1.3 (4)
S2—C3—C4—N2	−6.5 (4)	S1—C10—C13—C12	−0.8 (4)
C3—C4—N2—C6	177.7 (3)	C2—C12—C13—C10	1.4 (5)
C15—O1—C5—O2	23.2 (3)	C8—C9—C14—S2	−0.9 (4)
C15—O1—C5—C16	−94.4 (3)	C3—S2—C14—C9	0.6 (3)
C15—O1—C5—C17	141.4 (3)	C5—O1—C15—C6	−153.6 (2)
C4—N2—C6—C15	134.1 (3)	C5—O1—C15—C11	−32.6 (3)
C1—N1—C7—C11	140.1 (3)	N2—C6—C15—O1	−72.7 (3)
C4—C3—C8—C9	−179.6 (3)	N2—C6—C15—C11	173.2 (2)
S2—C3—C8—C9	−0.2 (3)	O2—C11—C15—O1	29.7 (3)
C3—C8—C9—C14	0.7 (4)	C7—C11—C15—O1	149.8 (3)
C2—S1—C10—C13	0.1 (3)	O2—C11—C15—C6	148.3 (2)
N1—C7—C11—O2	−70.8 (3)	C7—C11—C15—C6	−91.5 (3)

### Hydrogen-bond geometry (Å, °)

**Table:**

*D*—H···*A*	*D*—H	H···*A*	*D*···*A*	*D*—H···*A*
C9—H9···O1^i^	0.93	2.56	3.431 (4)	155
C8—H8···S2^i^	0.93	2.94	3.793 (3)	153
C12—H12···O1^ii^	0.93	2.68	3.466 (4)	143

Symmetry codes: (i) −*x*+3, *y*+1/2, −*z*+2; (ii) *x*−1, *y*, *z*.
